# The Mental Functions of the Brain

**Published:** 1902-12

**Authors:** 


**The Mental Functions of the Brain.** By BERNARD HOLLANDER, M.D. Pp. xviii., 512. London: Grant Richards. 1901.

In many ways this is an able book, and affords abundant food for thought and reflection. Some portions of the work are extremely interesting, especially those dealing with the early history of phrenology, about which the medical profession is undoubtedly too ignorant. The author has done good service in pointing out the debt we are under to Gall, the pioneer in the modern study of the anatomy and physiology of the brain, and an observer of the first rank in this field. He seems to have been a thoroughly scientific investigator, of extraordinary industry. Some of the plates reproduced from his great work are extremely good. There is good reason to credit Gall with the first clear description of aphasia, and of the exact part of the brain involved in this disorder. This was a discovery of no mean order in the state of knowledge at that time, and there can be no doubt that the study of the different forms of aphasia has thrown more light on the workings of the brain, and on the localisation of its functions, than almost any other. Gall made many other discoveries in the structure of the brain, of first-rate importance, and showed a really scientific mind in his indefatigability in collecting facts, and in his caution in forming theories from them. Unfortunately, the extravagant inferences of his followers, which appear to have never been countenanced by him, threw discredit on his researches, which, indeed, were in themselves so far in advance of his time, that it is not surprising that they met with strong opposition. The later absurdities of professional phrenologists still further obscured the value of his work: but it is to be hoped that at length due recognition, though so long delayed, will be awarded to him for the extent and value of his researches. The story of Gall's life should be a stimulus to every worker in science, as showing that, though sometimes long delayed, the value of good, honest work, must inevitably one day be recognised.

Whether the author is wise in seeking to revive the term "phrenology," seems to us doubtful: it has now so long been associated with pseudo-scientific "Professors," and in the popular mind with "bumps," that its rehabilitation in a better sense is practically impossible, and indeed is unnecessary, for we have in localisation of brain functions a term which, although cumbrous, is well understood, and has no such sinister connections.

After the discovery of the speech centres, that of the motor areas has been the most fruitful in advancing the knowledge of brain localisation. The study is one, however, of extraordinary difficulty, partly owing to the great complexity of the structure of the brain. Although the unravelling of this structure has proceeded apace during the last twenty years, due to improved methods of investigation, there is still a vast amount to be done; and until we have a far more exact knowledge of anatomy, we cannot advance far on the lines of differentiation of function.

That there must be localisation, or rather specialisation in the brain, to an extent unknown in any other organ is obvious from what we already know of it; indeed, it would be extraordinary if the most highly specialised organ in the body did not show the most advanced differentiation. Meanwhile, clinical observation on the effects of disease should form a most valuable aid to this study, and is capable of being much more utilised than it is at present. To be of permanent value, however, such observations must be exact, and in every case supplemented by *post-mortem* examination, which must include a careful microscopic study of the parts affected. To take, for instance, the case of tumours: no case of brain tumour can be safely utilised for purposes of localisation unless it is well circumscribed, exactly limited to one portion of the brain, and its effects on neighbouring tracts microscopically investigated.

In this book cases are inserted which are not full enough in clinical detail, are wanting in a *post-mortem* examination, or in which the pathological lesion was an extensive one. Such cases are of little value in an argument of the present kind.

A section of the book is taken up by an attempt to considerably extend the positive results hitherto worked out in brain localisation, by assigning certain forms of insanity to lesion of a special cerebral area for each. The author has collected a considerable body of evidence in favour of the localisations he brings forward for these diseases. Bearing in mind, however, that such localisations differ in kind from those now established by experiment and pathological observation in recent years, it is, perhaps, best at present to leave the question an open one. To make the position certain, the converse proposition, that those affections are never met with as a result of lesions of other parts of the brain, should be dealt with.

Further, it may be pointed out that if such cases can be definitely localised, they will not necessarily be coterminous with the external (anatomical) markings of the cortex.

We have said enough to indicate that the book may be read with profit by all medical men, that it will be found extremely interesting, and that the problems suggested by its perusal will stimulate thought, and lead to further endeavours to extend a branch of knowledge in which the near future promises a considerable advance.

